# Simultaneous Quantification of Three Polymorphic Forms of Carbamazepine in the Presence of Excipients Using Raman Spectroscopy

**DOI:** 10.3390/molecules190914128

**Published:** 2014-09-09

**Authors:** Marco Farias, Renato Carneiro

**Affiliations:** Department of Chemistry, Federal University of São Carlos, Rodovia Washington Luiz Km 235, São Carlos, SP 13565-905, Brazil; E-Mail: marco.ufscar@gmail.com

**Keywords:** carbamazepine, polymorphism, Raman spectroscopy, iPLS, multivariate calibration

## Abstract

The occurrence of polymorphic transitions is a serious problem for pharmaceutical companies, because it can affect the bioavailability of the final product. With several known polymorphic forms carbamazepine is one of the most problematic drugs in this respect. Raman spectroscopy is a vibrational technique that is becoming very important in the pharmaceutical field, mainly due to its highly specific molecular fingerprint capabilities and easy use as a process analytical tool. However, multivariate methods are necessary both for identification and quantification. In this work an analytical methodology using Raman spectroscopy and interval Partial Least Squares Regression (iPLS), was developed in order to quantify mixtures of carbamazepine polymorphs in the presence of the most common excipients. The three polymorphs CBZ I, CBZ III and CBZ DH (which is a dihydrate) were synthesized and characterized by PXRD and DSC. Subsequently, tablets were manufactured using excipients and 15 different mixtures of carbamazepine polymorphs. The iPLS model presented average prediction validation errors of 1.58%, 1.04% and 0.22% wt/wt, for CBZ I, CBZ III and CBZ DH, respectively, considering the whole mass of the tablet. The model presents a good prediction capacity and the proposed methodology could be used to perform quality control in final products.

## 1. Introduction

As described in the work of Bauer *et al*. [1]: “Polymorphism is the ability of a compound to exist in more than one crystal form with different unit cell parameters”. An active pharmaceutical ingredient (API) can exist in different polymorphic forms which present differences in some properties like melting point, chemical reactivity, apparent solubility, dissolution rate, and others [2,3]. The recognition of the importance of polymorphism in the pharmaceutical industry started in 1969 when the microscopist Walter McCrone published a review entitled *Pharmaceutical Applications of Polymorphism* [4]. However the polymorphic transition that occurred in the commercial product known as Norvir (ritonavir) was the culminating point that called the attention of the pharmaceutical industry and regulatory agencies to this issues, obliging them to review the quality control procedures implemented at the time [1,5].

The majority of the drugs are formulated and delivered in solid dosage forms [6] like tablets and capsules. A large variety of such drugs can display many polymorphic forms affecting the quality of the commercialized drug, especially when the dissolution rate is affected [7].

Among the drugs that exhibits polymorphic forms carbamazepine [8,9,10,11] is one of the most problematic compounds and it has a history of irregular drug performance and clinical failures [12]. A study conducted by Meyer *et al.* [13] demonstrated that the dissolution rates of carbamazepine tablets display high variability, even among tablets of the same brand. According to the biopharmaceutical classification system [14], carbamazepine is classified as Class 2, which means that this drug has low solubility in water and high permeability in human tissues. Therefore, the absorption of carbamazepine is determined by the rate of dissolution of the drug in the organism and any possibility of a polymorphic conversion in the tablet or capsule could seriously affect the solubility. A study by Kobayashi *et al.* [15] demonstrated that the dissolution rate of carbamazepine form I and dihydrate are lower than that of form III. The same study shows that the polymorph III of carbamazepine (commercialized in tablets) can convert into a dihydrate within two weeks and 40 °C under an atmosphere with 98% of relative humidity. Such information reveals the necessity to know what polymorphs are present in commercial tablets of carbamazepine.

The FDA guide for pharmaceutical solid polymorphism [2] contains several techniques to perform polymorphism characterization, and a review by Chieng *et al.* [16] suggests that at least two solid-state analytical techniques be used for characterization of a sample, but generally four techniques are employed. Among the techniques used to discriminate polymorphs, powder X-ray diffraction (PXRD) is the most used [17]. Nevertheless, this technique presents some problems when used to perform quantification of mixtures of crystalline forms due to its dependence on the particle size distribution (sometimes referred to as crystallinity). If the particles are too small, an enlargement of the peaks and a decrease of the PXRD signal will occur, and this is an unwanted characteristic for quantification. Sometimes there is the need for an API to have a particle size as small as possible, to increase its bioavailability, making the XRD unsuitable for this kind of analysis, although it is possible to determine carbamazepine polymorphs using XRD [18]. In commercial formulations (tablets) the API is mixed with excipients and compressed. The excipients also have different crystal habits and particle sizes that will yield a very complex diffractogram, presenting many peaks and decreasing the sensitivity of the technique to the API. In addition, the quantification problems using PXRD can be reinforced by the preferred orientation phenomenon [17].

Another analytical technique used to discriminate polymorphs is differential scanning calorimetry (DSC), which monitors thermal events related to polymorphic transitions. This technique can be used to characterize polymorphs because they present different melting points (and different enthalpies of fusion). However, there are two problems: the heat can induce polymorphic transitions during the DSC analysis, where the API can undergo conversion to different forms and; if there is a mixture of polymorphs, one of them can melt and solubilize the other forms, making it impossible to detect the melting points of these other forms [19,20]. Thus, DSC also presents disadvantages in the quantification of polymorphs in a mixture. If interconvertion or solubilization phenomena occur, the real polymorphic composition of a sample will be masked.

Spectroscopic techniques, such as infrared (IR), Raman and solid-state nuclear magnetic resonance (ssNMR), have shown to be highly successful in the analysis of physical characteristics. As already discussed by Bugay *et al.* [21], the choice of the analytical technique used for quantitation depends on its limitations and most of them are related to the sample preparation. Attenuated total reflectance (ATR) infrared spectroscopy is one of the techniques that need almost no preparation, as it is only necessary to place the sample in contact with the crystal. Nevertheless, this analysis only collects information from the surface of the sample in contact with the ATR crystal, in other words, the infrared radiation beam does not collect any information about the bulk sample. Another problem related to the use of IR in the analysis of commercial formulations is its own selection rules, because of the behavior of the excipients. Excipients generally have stronger absorptions than the API and the IR analysis results become difficult to interpret. This occurs because most excipients present bonds with high dipole moments. On the other hand, in Raman spectra the APIs have stronger signals than the excipients, which is an essential characteristic considering that in most cases, tablets contain more excipients than API. Raman is not sensitive to high dipole moment bonds, such as in most excipients (and water), but is highly sensitive to conjugated double bonds, aromatic rings and other structures present in most APIs. That is one of the reasons for choosing Raman as the analytical technique in this work.

However, Raman spectroscopy also has its limitations: the signal intensity can be influenced by the particle size [22] and colored samples can absorb the laser beam energy and heat up, yielding a low quality spectrum (due to the thermal background), burn the sample, or promote a polymorphic transition *in loco*. Finally, as the probe collects information from a very small area of the sample (nearly 1 mm^2^) [23], subsampling can be a problem [22,23]. Nevertheless, there are some procedures that can minimize the problems just quoted. Related to subsampling, it is possible to obtain an average spectrum using spectra from different positions in the sample. The problem with large crystals can be solved using a mortar. Such a procedure is only possible if the API does not transform into another polymorph upon applying pressure.

In short, the polymorphic structure can be analyzed at the molecular level (*i.e.*, with spectroscopic techniques) or at the particulate level (*i.e.*, using PXRD, thermal methods or microscopy). Nevertheless, the choice of the technique depends on the sample characteristics. If the sample is a tablet, it is necessary to consider that the API will be mixed with many excipients and generally the ratio between an API and excipients is low [21], hence the choice of the analytical technique must consider that analytical signal needs to be stronger for the API than for the excipients, which is most often true for Raman spectroscopy [6,24]. Moreover Raman spectroscopy can perform analyses without any sample preparation, even if the tablet is inside a closed transparent blister, thus avoiding sample preparation problems [23].

Previous studies show that is possible to quantify the mixture of the three carbamazepine polymorphs in raw materials using Raman spectroscopy [25,26]. However, even using the pure form III as raw material, polymorphic transitions can occur during the manufacturing process, and they need to be evaluated in the final product, when excipients are present. The polymorphic transition is more probable in wet granulation processes, where water is added to form granules and the mixture undergoes a drying step in a fluidized bed or by kiln-drying. In these cases, polymorphic transitions can occur by the formation of hydrates due to the water addition, and other polymorphs due to the heating in the drying step. In addition, even when direct compression is employed (without wet granulation), these polymorphic transitions can occur by the transference of moisture from the excipients to the API and due to the high pressure employed in the compression step.

In this study, Raman spectroscopy was used as an analytical tool to quantify mixtures of carbamazepine polymorphs (forms I, III and dihydrate) in the presence of excipients in laboratory-made tablets. For that, Partial Least Squares was used as a multivariate calibration model. In these tablets there was only 16.67% carbamazepine, which was a mixture of the three polymorphs. For the remainder of the tablets (83.33%) the most common excipients—microcrystalline cellulose, sodium croscamellose, magnesium stearate, colloidal silicon dioxide—were used. These lab-made tablets represent 50 mg commercial carbamazepine tablets.

## 2. Results and Discussion

Initially the polymorphs were characterized by PXRD and DSC [26]. The PXRD and DSC patterns confirmed the presence of CBZ I, CBZ DH and CBZ III according to the literature [26,27]. We prepared 15 samples using a defined proportion of excipients and different mixtures of the three polymorphs, following a ternary mixture design for calibration samples. The tablets were compressed and the Raman analyses were performed. More information is presented in the Experimental Section.

After the acquisition of Raman spectra, two preprocessing tools were used: the spectra were normalized to the area to cancel the influence of signal intensity among the samples and; the spectra were derived (first derivative). The first derivative preprocessing increases the data quality because one peak gives rise to two peaks, aiding to differentiate very close peaks and increasing the quality of the model [28], that in turn helps in the model building. An interval Partial Least Squares regression model was built, as this model uses only the most discriminative range of variables in the spectra, and it is a valuable way to build models because it eliminates nonlinear variables, noise, and variables which are not related to the discrimination of the polymorphs.

To build the calibration model, it is necessary to know how many latent variables are necessary to get the best model. The procedure used to calculate the ideal number of latent variables was cross-validation (leave-one-out). In this procedure, one sample is left out of the calibration set and the sample left out is then predicted [28], the procedure is repeated until all samples have been left out and predicted. The best model will present a small error and will not consider an excess of latent variables to avoid an over-fitted model. The error is calculated using the root mean square error of cross-validation (RMSECV), given by Equation (1):

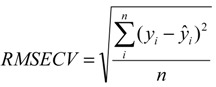
(1)
where *n* is the number of samples, *y_i_* and *ŷ_i_* are the real and predicted concentration of a polymorph in the sample *i*.

**Figure 1:**
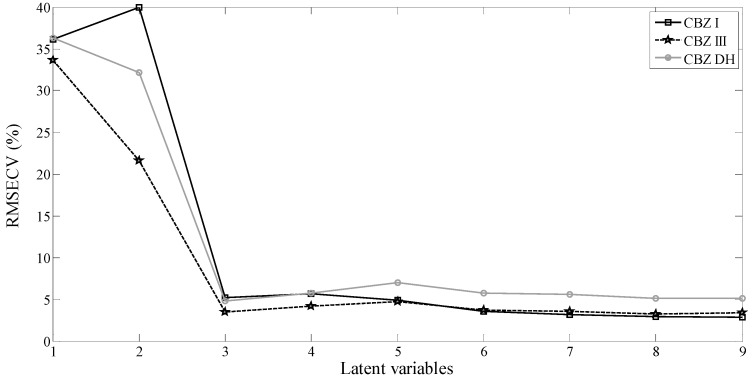
Distribution of the variance per latent variables.

Figure 1 shows the RMSECV *vs.* the number of latent variables. In this figure it is possible to see that three latent variables are sufficient to build the ternary calibration models. In addition, the first three latent variables explain 99.59% of the total variance of the data. It was expected that three latent variables would be enough because the only source of variation in the Raman spectra were the three polymorphs, since the concentration of excipients was constant.

**Figure 2:**
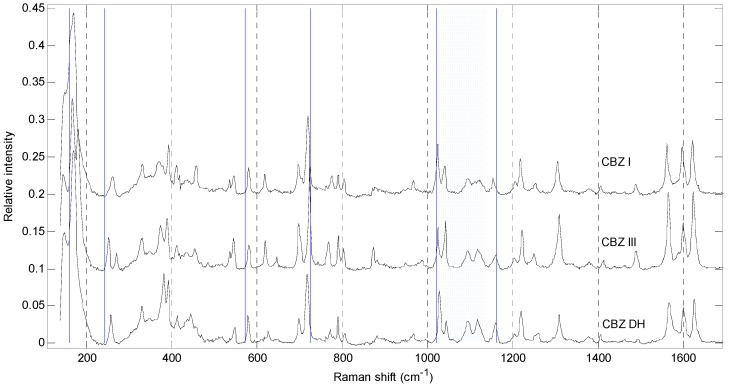
Raman spectra of pure samples with excipients: (a) CBZ I, (b) CBZ III, (c) CBZ DH. In blue, the ranges selected by the best iPLS model, in cm^−1^: 160 to 244; 572 to 726; and 1021 to 1162.

Figure 2 shows the spectra of tablets obtained using pure polymorphs. The peaks in the ranges 160–250 cm^−1^, 700–800 cm^−1^ and 1000–1100 cm^−1^, present a unique pattern, demonstrating that each one of the pure samples of CBZ I, CBZ III and CBZ DH have unique spectra, however, this fact does not imply that iPLS models need to choose exactly these ranges. The quantification using iPLS is possible because of the unique spectra of the polymorphs and because the variation among the spectra is directly related to the proportion of the mixture of polymorphs in the tablets.

**Figure 3:**
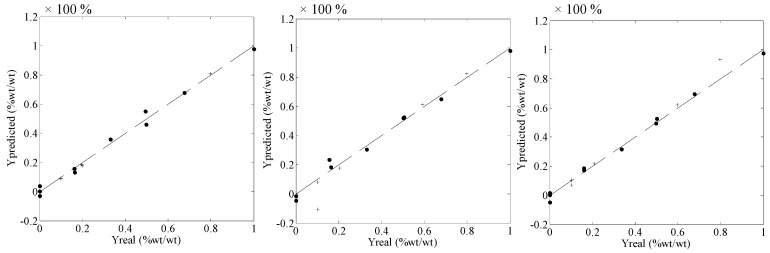
Real *vs.* predicted values for CBZ DH (**left**), CBZ I (**middle**) CBZ III (**right**). The percentage values consider the mass of a polymorph related to the total mass of carbamazepine. The straight line is the exact correlation between real *vs.* predicted values.

Notes: The symbol (+) refers to the samples of validation and the dot (∙) refers to the calibration samples.

Figure 3 shows the very good correlation between real values and values predicted by the iPLS models for the three polymorphs. This figure contains all the calibration and validation samples and the coefficients of determination (R^2^) of these correlations are presented in Table 1. In addition, Table 1 shows the root mean square errors of calibration (RMSEC), validation (RMSEV) and cross-validation.

CBZ I presented the highest (worst) RMSEV (9.49%). As can be seen in Table 1, the comparison between the RMSECV and RMSEV values shows good agreement, demonstrating that the model is capable of predicting new samples. The R^2^ values demonstrate that the residual values for the models are very low, meaning that the predicted values are very close to the real values and the model works fine.

**Table 1:** Parameters for iPLS model for Raman spectroscopy (% wt/wt).

Parameters	Polymorphs		
	CBZ I	CBZ III	CBZ DH
RMSEC	3.38 (0.56)	2.30 (0.38)	3.02 (0.50)
RMSECV	5.19 (0.86)	3.49 (0.58)	4.82 (0.80)
RMSEV	9.49 (1.58)	6.27 (1.04)	1.34 (0.22)
R^2	0.965	0.994	0.999

Notes: intervals used in the model: [160–244, 572–726 and 1021–1162] cm^−1^; Preprocessing: Normalized per area, 1st derivative; Errors consider the mass of a polymorph related to the total mass of carbamazepine; In brackets the errors which consider the mass of a polymorph related to the total mass of the tablet.

The RMSEV values for CBZ I (9.49%) and CBZ III (6.27%) were considerably larger than that for CBZ DH (1.34%), as shown in Table 1. This difference was caused due to the high validation error for just one sample of each model: the first validation sample for CBZ I; and the last validation sample for CBZ III. This can be noticed in Figure 3 (middle and right). However, the error could be smaller if more spectra were obtained from each sample (in this work 10 spectra/sample were obtained). In addition, the problem can occur due to lack of homogeneity of the polymorphs in these two samples.

In the lab-made tablets, the total concentration of carbamazepine was 16.67% (50 mg of carbamazepine for 250 mg of excipients). Thus, if the whole mass of the tablets is considered, the highest validation error will be 1.58% (wt/wt) (for CBZ I). Then, in the presence of the excipients for a tablet of 50 mg of carbamazepine, it will be possible to predict the concentration of a specific polymorph with a standard error near 2%. It is worth noting that it is the highest error of validation of the models.

## 3. Experimental Section

### 3.1. Preparation of Polymorphs

Polymorph III (CBZ III) was kindly provided by EMS Pharmaceutical Company (Hortolândia, Brazil). Polymorph I (CBZ I) was prepared weighing 1 g of CBZ III and leaving it in an oven at 170 °C for two h. The dihydrate (CBZ DH) was prepared weighing 1 g of CBZ III and adding ultrapure water until total immersion of the sample, following by drying at room temperature for four days [26].

### 3.2. Characterization of the Polymorphs

The three carbamazepine polymorphs were characterized using Raman, DSC and PXRD. The X-ray diffractometer was a RigakuDmax 2500PC (Tokyo, Japan) with a copper source (wavelength of 0.154 nm) with voltage of 40 kV and emission current of 40 mA. The samples were analyzed from 5° to 45° (2θ) at a scan rate of 0.2° min^−1^. For thermal analyses a Shimadzu DSC-60 (Kyoto, Japan) was used, with a heating rate of 10 °C min^−1^ under helium atmosphere and temperature range from 30 °C to 220 °C. For Raman analysis a B&WTek *i*-Raman spectrometer model BWS 415–785H (Newark, NJ, USA) was used coupled to a BAC151 microscope, with a 785 nm laser, spectral resolution of 3.5 cm^−1^, spectral range of 52–1708 cm^−1^, 30 s of acquisition, laser power 31.6 mW and a 20× objective lens.

### 3.3. Preparation of the Tablets

For the preparation of the tablets, information about the excipients used in typical carbamazepine tablets was collected from a group of package insert of medicines commercialized in Brazil. There are four common excipients used in all formulations evaluated: microcrystalline cellulose, sodium croscamellose, colloidal silicon dioxide and magnesium stearate. The proportion of the excipients is not mentioned in the package inserts so the *Handbook of Pharmaceutical Excipients* [29] was consulted to find the accepted proportions in a formulation, for each of the excipients. In this work 16.67% of carbamazepine (mixture of polymorphs) was used and the proportions of excipients were: microcrystalline cellulose (74.17%), sodium croscamellose (4.17%), colloidal silicon dioxide (0.83%) and magnesium stearate (4.17%). Each tablet has a total of 300 mg, where 250 mg are excipients and 50 mg are carbamazepine polymorphs.

The polymorphs were added in each tablet according to a ternary design, totaling 10 calibration samples and another five validation samples, according to Table 2. All the samples were homogenized using a mortar and pressed using a 13 mm evacuable die and a pressure of 1 ton.

**Table 2:** Amount of CBZ in mg of each polymorph for the calibration (1–10) and validation (11–15) samples.

Samples	Polymorphs		
	CBZ I	CBZ III	CBZ DH
1	49.4	0	0
2	0	50	0
3	0	0	50.8
4	25.3	25.2	0
5	25.2	0	24.7
6	0	25.1	24.9
7	34.3	8.1	8.2
8	7.9	34.3	8.3
9	8.2	8	33.9
10	16.4	16.2	16.4
11	40.2	5.2	5
12	5.1	40	5
13	5.1	5.1	40.1
14	30	10.6	10.1
15	10.2	29.8	9.8

### 3.4. Acquisition and Treatment of the Data

Ten Raman spectra were obtained of each sample, from different points on the tablet surface. These spectra were used to calculate a representative average spectrum for each sample. This procedure is necessary to minimize problems related to the subsampling due to the small spot of the laser.

The average spectra of each sample were normalized and the first derivative was obtained. The spectra from the calibration samples were used to build the regression model and the prediction samples, used to verify the power of prediction of the regression models. The iPLS models were built using the PLS_toolbox 6.2 (Eigenvector Research Inc., Wenatchee, WA, USA), available for Matlab^®^ 2011a (Mathworks Inc., Natick, MA, USA).

## 4. Conclusions

Raman spectroscopy allied with multivariate calibration yield low errors in the prediction of mixtures of carbamazepine polymorphs and its most common excipients, with errors around 2% related to the total mass of the tablet. Considering that commercial formulations can have exactly this content, or a little different, this approach shows good potential to be an analytical technique used to test commercial formulation. Such an application could be very attractive to the pharmaceutical industry and regulatory agencies considering the good results obtained and the portability of some Raman equipment. Specifically for the case of carbamazepine tablets, where the relation between API and excipients is higher (there are tablets with 100 mg and 200 mg of Carbamazepine) the model could work quite well.
